# High prevalence of *Entamoeba moshkovskii* infection in HIV seropositive patients of Barak Valley, Assam, India

**DOI:** 10.1186/1471-2334-14-S3-P1

**Published:** 2014-05-27

**Authors:** Joyobrato Nath, Sankar K Ghosh, Prithwiraj Bhattacharjee, Jaishree Paul, Baby Singha

**Affiliations:** 1 Department of Zoology, Gurucharan College, Silchar, Assam, India; 2 Department of Biotechnology, Assam University, Silchar, Assam, India; 3 Department of Medicine, Silchar Medical College and Hospital, Silchar, Assam, India; 4 School of Life Sciences, Jawaharlal Nehru University, New Delhi, India

## Background

It is now well established that *Entamoeba moshkovskii* and *Entamoeba dispar* are two distinct non-pathogenic species, microscopically indistinguishable from pathogenic *Entamoeba histolytica*. Being endemic, there are no data on the prevalence of these commensal infections from the North Eastern part of India.

## Methods

A total of 274 stool samples collected in this cross sectional study from HIV seropositive patients attending the ART centre of SMCH, Assam, India were screened for cyst and/ trophozoite stage of Entamoeba using iodine staining technique and then subjected to *SSU* rRNA gene based multiplex PCR assay.

## Results

Out of 274 stool samples, multiplex PCR assay of 61 microscopy positive samples showing cyst and/ trophozoite stage of Entamoeba, revealed a higher prevalence of *E. moshkovskii* (12.8%; 95% CI= 9.30, 17.28) and lower prevalence of *E. dispar* (6.2%; 95% CI=3.85, 9.77) compared to *E. histolytica* (8.1%; 95% CI= 5.31, 11.91; *p*< 0.05). Of the 61 samples that were microscopically positive, 5 were *E. histolytica*, 8 were *E. dispar*, 25 were *E. moshkovskii*, 17 were mixed infections with *E. histolytica* and 6 were PCR negative which may be non-histolytica/dispar/moshkovskii infection. Thus, only 36% of the 61 microscopy positive stool samples were actually *E. histolytica*, implying that remaining 64% of so called infections were over represented and would have been treated unnecessarily.

## Conclusion

This is the first report of *E. moshkovskii* infection from this region of India and its high prevalence signifies an urgent need of amoebiasis diagnosis using advanced molecular tools to avoid overrepresentation and unnecessary chemotherapy.

